# Nuclear Functions of the Tyrosine Kinase Src

**DOI:** 10.3390/ijms21082675

**Published:** 2020-04-11

**Authors:** Giulia Bagnato, Martina Leopizzi, Enrica Urciuoli, Barbara Peruzzi

**Affiliations:** 1 Multifactorial Disease and Complex Phenotype Research Area, Bambino Gesù Children’s Hospital, IRCCS, 00165 Rome, Italy; giulia.bagnato@outlook.it (G.B.); enrica.urciuoli@opbg.net (E.U.); 2 Department of Medico-Surgical Sciences and Biotechnology, Polo Pontino, Sapienza University, 04100 Latina, Italy; martina.leopizzi@uniroma1.it

**Keywords:** Tyrosine phosphorylation, Src-family kinases, subcellular localization, nucleus, oncogenes

## Abstract

Src is the representative member of the Src-family kinases (SFKs), a group of tyrosine kinases involved in several cellular processes. Its main function has been for long confined to the plasma membrane/cytoplasm compartment, being a myristoylated protein anchored to the cell membrane and functioning downstream to receptors, most of them lacking intrinsic kinase activity. In the last decades, new roles for some SFKs have been described in the nuclear compartment, suggesting that these proteins can also be involved in directly regulating gene transcription or nucleoskeleton architecture. In this review, we focused on those nuclear functions specifically attributable to Src, by considering its function as both tyrosine kinase and adapting molecule. In particular, we addressed the Src involvement in physiological as well as in pathological conditions, especially in tumors.

## 1. Introduction

**Figure 1:**
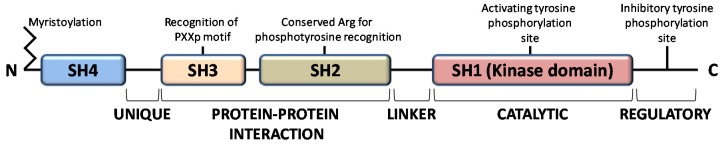
Domain structure of Src family kinases.

The members of the Src family of protein tyrosine kinases (SFKs) are expressed in all mammalian cells, where they are implicated in pivotal physiological cellular processes as proliferation, migration, differentiation and survival, as well as in pathological cancer onset and progression, when overactivated. These are non-receptor tyrosine kinases that, once activated by external stimuli acting on receptors for growth factors, cytokines, steroid hormones, on G protein-coupled receptors and adhesion proteins, start a signaling cascade leading to widespread effects [1]. The family consists of eleven members, among which Src is the prototype enzyme: the members of the main group (Src, Yes, Fyn, Fgr, Blk, Hck, Lck, and Lyn) are closely related, while Frk, Srm, and Brk constitute a more distantly related group. Among them, Src, Yes, and Fyn are expressed in mammals in a ubiquitous manner, while the expression pattern of the other members is tissue and/or cell restricted [2]. These SFKs share high homologous structure consisting of four consecutive Src Homology (SH) domains: an SH4 membrane-targeting region at their N-terminus, that can be myristoylated and/or palmitoylated to allow membrane localization; an intrinsically disordered unique domain, which exhibit strong sequence divergence among SFK members; the regulatory SH-2 and SH-3 domains precede a large catalytic C-terminal domain (SH1) with the hallmark of Src kinases, an autoinhibitory phosphorylation site that is the Y527 residue in human Src (Figure 1) [3].

All members of the Src family kinases present with myristoylation at the N-terminus [3]. Myristoylation is an irreversible modification that occurs cotranslationally and is catalyzed by the *N*-myristoyl transferases (NMTs). The 14-carbon myristoyl group attached to a glycine residue of the SH4 domain is necessary but not sufficient to anchor SFKs to the plasma membrane, given that a second signal is required. For the other SFKs, a palmitoylation motif functions as the second signal to be targeted to the membrane, while Src requires a polybasic cluster of amino acids to interacts with the inner leaflet of the membrane bilayer. Recent evidence demonstrates that dimerization as well as kinase activity and substrate phosphorylation are mediated by the first domains along with the N-terminal myristoylation [4,5,6]. Le Roux and coauthors performed a kinetic characterization of the Src binding to the lipidic layer of the plasma membrane, demonstrating that the first N-terminal domains of myristoylated Src are involved in the formation of a stable dimer whose membrane binding is much stronger than the monomeric Src form. Interestingly, the equilibrium between monomeric (labile) and dimeric (persistent) form of myristoylated Src can regulates Src localization and the downstream Src signaling at specific membrane sites [4,7,8]. 

The function of SH2 domain is to specifically bind a target protein by its phosphorylated tyrosine residue within a longer peptide motif, thus allowing SH2 domain-containing proteins to interact. The recognition of the phospho-tyrosine residues within the SH2 domain is guaranteed by a universally conserved arginine residue needed to form the proper electrostatic interactions with the phosphorylated tyrosine [3].

The SH3 domain is crucial for protein-protein interaction by mediating assembly of specific protein complexes, typically via binding to proline-rich peptides bearing the ‘PxxP’ motif in their respective binding partner [9]. 

The catalytic SH1 domain at the C-terminus contains the site of activating tyrosine phosphorylation, residue Y419 in human Src kinase. This domain, the most conserved in all tyrosine kinases, contains an ATP-binding pocket and the tyrosine-specific protein kinase activity [10]. 

Inactive Src is maintained in a closed conformation, in which the SH2 domain is engaged with the phosphorylated autoinhibitory residue, the SH3 domain binds the SH2-kinase linker sequence and the activating-tyrosine residue is dephosphorylated. Indeed, it has been demonstrated that the inactive conformation of the kinase domain is energetically more favorable when it is not phosphorylated. The work by Xu et al. on the crystal structure of Src demonstrated that the unphosphorylated activation segment adopts an α-helical structure that contributes to stabilize the closed conformation of Src. Therefore, their hypothesis is that any effector interaction that disrupts this helical structure would bring about the relief of negative constraint and make the enzyme temporally active [11]. In this context, the adaptor protein Shc seems to be involved in the structural changes required to activate Src. Indeed, Shc can bind Src when its activation segments are unphosphorylated, inducing a structural alteration of the activation segment conformation leading to the relief of the negative constraint of the catalytic domain. This event allows the autophosphorylation of the activation segment, thereby guarantee the stabilization of the catalytic domain active conformation [12]. Therefore, the dephosphorylation of autoinhibitory tyrosine disrupts its intramolecular interaction with the SH2 domain, leading to an open conformational state that allows autophosphorylation of activating-tyrosine residue, resulting in Src activation [13,14,15,16]. 

Once autophosphorylation has occurred, the activation of the kinase domain required large rearrangements in its orientation. Autophosphorylation displaced the regulatory domains that become more flexible and establish a strong cross-talk with the kinase domain, which in turn gains rigidity, leading to the stabilization of the ATP binding site [13].

By modulating the phosphorylation status of the SFK inhibitory tyrosine residue, several tyrosine kinases and phosphatases are involved in the fine-tuning regulation of Src and other kinase activation. Indeed, phosphorylation of Y530 can be removed by several protein phosphatases, thereby function as activators of Src, such as protein tyrosine phosphatase-α (PTPα), PTP1, SH2-containing phosphatase 1 (SHP1) and SHP2 [17]. The upstream signals involved in the activation of such protein phosphatases seem to be cell-specific. For example, PTP1B, a ubiquitously expressed protein phosphatase, is involved in dephosphotylating Src pY530 in breast cancer cell lines but not in the normal cell counterpart [18]. On the other hand, the non-receptor tyrosine kinase Csk serves as an indispensable negative regulator of the SFKs by specifically phosphorylating their negative regulatory site, thereby suppressing their activation. The activation of Csk depends on several upstream mechanisms, the first of which is the membrane anchoring mediated by scaffolding proteins, since Csk lacks the transmembrane domain allowing the anchorage to the lipidic bilayer, where the most of SFKs reside [19].

Src and the other tyrosine kinases of the family are downstream targets for cell surface receptors, and function as a link between the membrane receptors and the cytoplasmic signaling machinery, thereby regulating many fundamental cellular processes, including cell growth, differentiation, cell shape, migration and survival, and specialized cell signals [2]. In this context, it is worth mentioning that, although the ubiquitous expression of Src, the specific deletion of its gene in an animal model (Src knock-out mice) leads to a peculiar bone osteopetrotic phenotype [20], highlighting the crucial role of this tyrosine kinase in the cells of the bone tissue, both on osteoclast [21,22] and on osteoblast side [23], and the evidence that the other SFK members are able to vicariate the lack of Src in the other tissues. 

## 2. Nuclear Functions of SFKs other than Src

The main functions exerted by SFKs are related to their membrane and cytoplasmic localizations. As downstream targets of receptor tyrosine kinases (RTKs), SFKs affect cell proliferation via the Ras/ERK/MAPK pathway and regulate gene expression and angiogenesis via transcription factors such as STAT molecules. In their cytoplasmic functions, SFKs can interact with integrins, actins, GTPase-activating proteins, scaffold proteins such as p130CAS and paxillin, and kinases such as focal adhesion kinases, thereby affecting cell adhesion and migration [1]. Beside these membrane/cytoplasmic functions, SFKs have been described in other subcellular compartments, as the nucleus, the Golgi apparatus, late endosomes/lysosomes and mitochondria [24,25,26,27,28]. Subcellular distribution of SFKs other than Src are reported in the Table 1. 

Matsushima and coauthors have demonstrated a nuclear function for the tyrosine kinase Fyn in cardiomyocytes. Among the nicotinamide adenine dinucleotide phosphate (NADPH) oxidases, the main sources of reactive oxygen species (ROS) in the cardiovascular system [41], NOX4 expression and activity is fine tuning regulated in cardiomyocytes, playing a crucial role in the development of cardiac remodeling and injury. Authors showed that Fyn, once activated by oxidative stress, binds the c-terminal of NOX4 and colocalizes with it in perinuclear mitochondria, endoplasmic reticulum and nucleus. Doing this, Fyn serves as a negative feedback regulator of NOX4 in cardiomyocytes during cardiac remodeling [31]. 

Among SFKs, also Brk (human breast tumor kinase) and its orthologue Sik (mouse Src-related intestinal kinase) have been described to exert some nuclear functions. These tyrosine kinases are distantly related to the Src family, having a similar structure but lacking the myristoylation signal. Prior to Derry et al. work, no substrates of Sik and Brk had been identified. Authors demonstrated that Sam68 (Src associated in mitosis; 68 kDa), a RNA- binding protein that was first identified as a major target of Src during mitosis [42], can be phosphorylated by Brk/Sik within the nucleus, thereby negatively regulating its RNA binding activity [37]. Therefore, these data showed that, in addition to Sam68 phosphorylation by SFKs during mitosis, Brk/Sik can phosphorylate Sam68 and regulate its activity within the nucleus during the rest of the cell cycle. 

Among the SFKs exerting nuclear functions, the Lyn tyrosine kinase is known to be involved in the cellular response that includes cell cycle arrest, activation of DNA repair, and, in the event of irreparable damage, induction of apoptosis [43]. The work by Yoshida and coauthors describes Lyn involvement in the induction of the stress-activated protein kinase (SAPK), and that this pathway is functional in the induction of apoptosis by genotoxic agents [38]. 

In the context of cellular response to DNA damage, Rak tyrosine kinase has a peculiar role given that, unlike Src and the most of the other SFKs, it functions as a tumor suppressor in human cancer [39]. Indeed, it has been demonstrated a critical role of Fyn-related kinase (Frk)/Rak in the maintenance of genomic stability, at least in part, through protecting BRCA1 [40].

## 3. Src Translocation into the Nucleus

Some Src family tyrosine kinases have been described to reside in the nucleus, although there is a lack of nuclear localization signal (NLS). The NLS is a short sequence of positively charged lysines or arginines exposed on the protein surface that “tags” a protein for import into the cell nucleus by nuclear transport [44]. Canonical NLS are not present on SFK amino acid sequence, thus suggesting that these proteins may enter the nucleus through a not-canonical NLS or by an alternative way from active transport. In 1993, David-Pfeuty and coauthors suggested the intriguing hypothesis that nonmyristoylated proteins can readily accumulate into the nucleus, thereby attributing to myristoylation a role in preventing unregulated nuclear transport of proteins. They also raise the possibility that, in specific circumstances, a subfraction of Src may translocate into the nucleus where it exerts peculiar functions, thus behaving like its nonmyristoylated counterpart [45]. 

In support of David-Pfeuty hypothesis, we recently demonstrated that Src nuclear localization in osteoblasts and osteosarcoma cell lines is related to the myristoylation status of the cells. Indeed, low aggressive osteosarcoma SaOS-2 cells show high content of nuclear Src with a low myristoylation and low expression of N-myristoyltransferase (NMT) enzymes, in comparison to high metastatic 143B osteosarcoma cells, in which nuclear Src is lower while myristoylation and NMT expression is very high [46]. 

An intriguing hypothesis about the complex relationship between Src myristoylation and its subcellular localization raises from the work of La Roux and coworkers [47], in which they discovered a myristoil-binding site in the SH3 domain. The N-terminal myristoyl group can bind to this SH3 binding site when Src is not anchored to the lipid layer, therefore the interaction of the myristoyl group with lipids may prevent nuclear localization. Thus, intramolecular interactions involving SH3 -mediated sequestering of the myristoyl group may be relevant in the context of Src nuclear localization [47].

## 4. Physiopathological Roles of Nuclear Src

Although tyrosine kinases are well known to function as signaling molecules downstream of extracellular stimuli at the plasma membrane, some SFKs have been described to reside in the nucleus where they regulate tyrosine phosphorylation of nuclear proteins, and/or function as cofactor in multiprotein complexes [48]. Therefore, the roles exerted by Src in the nucleus could be dependent or not on its catalytic activity. Indeed, beside its capability to phosphorylate tyrosine residues on target proteins, the SH2 and SH3 domains in the Src structure are involved in protein-protein interaction that can be independent from Src activation status. In particular, nuclear Src seems to exacebate the activity of oncogenes, and to counteract the protecting function of oncosuppressor, in general by inducing their nuclear export. Here we reviewed the main mechanisms involving Src nuclear functions.

### 4.1. Regulation of Gene Transcription and Chromatin Architecture

Changes in the structure of nuclear compartment are frequently observed during transcription, cell differentiation, senescence, cell cycle and tumorigenesis [49], and evidence of active nuclear Src has been reported in different contexts. A study carried out on NT2D1 non-seminoma fibroblasts reveals that Src phosphorylation is constitutively present in the nuclei of these cells, representing a downstream effector of c-MET pathway [50]. c-MET is the membrane receptor of HGF (Hepatocyte Growth Factor). HGF can increase the aggressive and malignant behavior of NT2D1 cells through c-MET activation [51]. The inhibition of Src deletes the HGF-dependent increase of cell proliferation rate, migration and cell invasion. c-MET recruits Src when activated by HGF, and this stimulus seems to be a key point allowing Src to translocate into the nucleus where it interacts with some gene promoters. In this context, a pivotal role is played by the cancer microenvironment, given that in the culture basal conditions (without administration of HGF) the inhibition of Src causes the augment of invasiveness but decreases the cell proliferation rate and migration capability of mouse NT2D1 fibroblasts independently from c-MET pathway, may be due to the Src recruitment by other homeostatic pathways controlling the aggressiveness of these cells [50]. 

**Figure 2:**
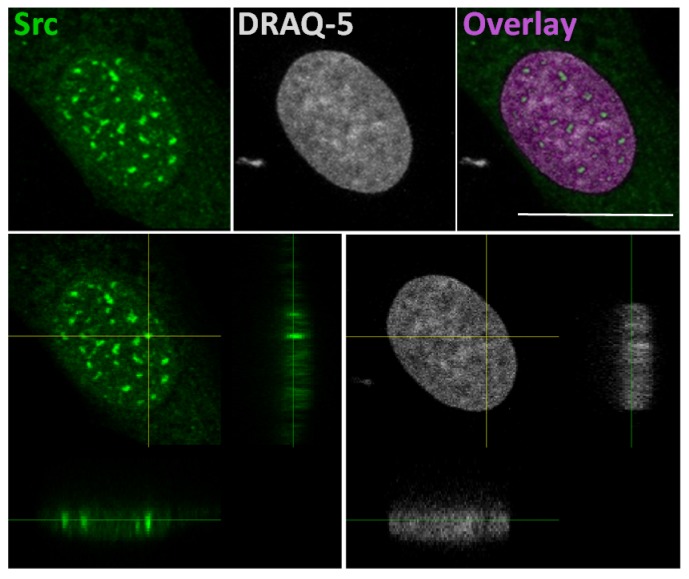
In the upper panels: representative confocal microscope images of immunofluorescent staining for Src (left panel), nuclear dye DRAQ-5 (middle panel) and the overlay fluorescence (right panel - pseudo-colored in purple) of detailed nuclei of human osteosarcoma cell line SaOS2. Scale bar: 20 μm. In the lower panels: confocal microscope average XYZ projection of a stack of images of Src (left panel) and DRAQ-5 (right panel) fluorescences. The yellow cross-section pinpoints the location for the YZ and XZ axes projections.

The idea that Src could interact with gene promoters is based on a study that explains a correlation of SFKs with the chromatin structural changes observed following growth factors stimulation [52]. In this study, authors developed a pixel imaging technique of the nucleus to quantitatively detect changes of chromatin structure and condensation levels. They demonstrated that SFK activation by serum-conveyed growth factors localize into the nucleus more frequently in the euchromatin than the heterochromatin areas, and that their kinase activity is required for the chromatin organization, given that growth factor stimulation effects are avoided in mouse embryonic fibroblast SYF cells, which are genetically deficient in expression of Src, Yes, Fyn and Lyn tyrosine kinases. Taken together, this evidence suggested that the SFKs could be useful to create an “open” chromatin more accessible to transcriptional factors [52]. In this context, we recently demonstrated the Src nuclear localization in osteoblasts and low aggressive osteosarcoma cells [46], and in particular we observed nuclear Src accumulation in hypocondensated chromatin, as demonstrated by the low DRAQ5 staining (Figure 2). This finding, together with the work by Takahashi, strongly suggests a function for nuclear Src in the regulation of transcription. 

As regards cancer cells, the protein p300, a large histone acetyltransferase with the function of coactivator, was at first known to be a tumor suppressor but the recent discovery of *p300* gene mutations seems to suggest a role for this enzyme in the oncogenic transformation [53]. In the tumor pancreatic environment, p300 seems to interact with Src, which can in turn activate the pro-migratory genes such as *HMGA2* and *SMYD3* [54]. The binding of Src and p300 to the sequence of DNA depends on chromatin and cell-type background. In those cancers in which Src has been found downregulated, the clinical trials based on Src-inhibitor therapy have proven to be ineffective and data by Paladino et al. provide some explanation about these failing therapies, as Src seems to be more involved in the migratory pathway than in survival signaling. Although these works describe some peculiar roles of Src in specific micro-environment, Src remains a good therapeutic target to prevent tumor metastasis [55].

### 4.2. Src-Dependent Regulation of Tumor Suppressors

As an example of its catalytic-dependent and independent nuclear functions, Src is able to regulate the localization of INhibitor of Growth 1 (ING1) from nucleus to cytoplasm through phosphorylation-dependent and independent mechanisms, thus contributing to alter the capability of ING1 to induce apoptosis. ING1 plays a role in epigenetic regulation as tumor suppressor, being a stoichiometric member of histone acetlytransferase (HAT) and histone deacetylase (HDAC) complexes. When Src expression and/or activation is altered, as in many types of cancer, the ING1 levels are deregulated accordingly, and decreases following Src activation. Src destabilizes ING1 by phosphorylation, thereby inducing its export from nucleus. The Src phosphorylation-independent mechanism is based on the capacity of Src to bind directly ING1: in this role as cofactor, Src may prompt the degradation of ING1, or, as an alternative, kinase-dead Src may recruit and/or activate other tyrosine kinases to target this tumor suppressor [56]. 

Another protein that can be altered by Src-dependent kinase activity is the Runt domain transcription factor 3 (RUNX3). RUNX3 is a transcription factor known to be a tumor suppressor involved in proliferation, apoptosis and cellular differentiation. Oxidative stress causes RUNX3 mislocalization in cytoplasm in colon cancer cells. In conditions of oxidative stress, both Src expression and activation is positively regulated in the nucleus by HDAC1, known to involved in the transcription of oncogenes [57,58] and active Src phosphorylates RUNX3 leading to its cytoplasmic localization [59]. 

### 4.3. Src and Estrogen Receptor

Studies on the subcellular localization of steroid receptors have demonstrated that they can have effects other than the non-genomic action, thereby revealed their ability to interact with target effectors and activate signaling pathways. Src is involved in the regulation of estrogen receptors, which are known to regulate the homeostasis of a variety of tissues, including the bone [60]. Low levels of estrogen deficiency lead to accelerated bone loss and this is the primary cause of postmenopausal osteoporosis [61]. Estrogens are also responsible for an anti-apoptotic effect in osteoblasts [62]. Further studies have demonstrated that Src interacts with the estrogen receptor even in other cells such as the uterine cells and human breast cancer cells. Indeed, in the nuclei of uterine cells, active Src can phosphorylates estrogen receptor α (ERα) and enhances its transcriptional activity due to the activity of SHP2 (Src-Homology Protein2) [63]. SHP2, a protein encoded by the gene *PTPN11*, is generally located in the cytoplasm, but it is also known to translocate in the nucleus when DNA damage occurs [64]. SHP2 enhances Src tyrosine kinase activity by removing its inhibitory phosphorylation and Src, in turn, phosphorylates ERα, thus allowing its binding to the progesterone receptor promoter and driving its transcription [63]. 

Instead, the study of Castoria and colleagues demonstrates that in the breast cancer tumor environment, Src can promote the tumor progression through its tyrosine kinase activity [65]. The Tyr 537 residue of ERα is a key regulatory site for its activity and localization, and also connects ERα with Src [66]. The stimulation with estradiol promotes Src activity and leads to the phosphorylation of ERα in Tyr537, thus driving the nuclear export of the receptor and regulating hormone responsiveness of DNA synthesis in breast cancer cells [65]. 

### 4.4. Interaction with the Nuclear Envelope Protein Emerin

Emerin is a nuclear inner membrane protein whose gene mutations are related to Emery-Dreifuss Muscular Dystrophy, an X-linked disease [67]. Tifft and coworkers demonstrated that emerin function is regulated by several tyrosine kinases, including Her2, Src and Abl. In particular, Src can mediate the signaling of Her2 by phosphorylation of three specific tyrosine residues in human emerin: Y59, Y74 and Y95 [68]. These three amino acid residues could not be the only residues phosphorylated by Src, since even the Y4, Y34, Y41, Y105 and Y155 are predicted Src-target sites [69]. Tifft and colleagues demonstrated that the substitutions of the tyrosine with phenylalanine, in the sites recognized by Src, reduced the capability of emerin to bind BAF (barrier-to-autointegration factor, also known as BANF1), a conserved chromatin regulator that also binds lamins. Emerin binds proteins that are crucial for the spatial organization of centrosome and nuclear structure, influences the actin cytoskeletal dynamics and helps to fasten silent chromatin [70]. Emerin is also involved in the mechano-transduction signaling, as it has been described as a downstream detector of mechanical stress. In more detail, emerin binds Lamin A, another nuclear envelop protein, and emerin depletion leads to an increased nuclear rigidity hindering the nuclear adaptation to mechanical forces. Guilluy and colleagues showed that the phosphorylation of Y74 and Y95 of emerin residues by Src mediates the mechanical adaptation of nuclei to mechanical force [71]. Some recent evidence demonstrate that the cells cultured on soft matrices induced emerin phosphorylation and the mislocalization of nuclear envelope proteins in the nucleoplasm [72]. The authors also suggest that emerin is able to reorganize the chromosome territories in cells on softer matrix and they speculate that emerin phosphorylation acts as an upstream regulator of lamin localization resulting in substantial changes of the transcriptional regulation in a substrate stiffness-dependent manner [72].

### 4.5. Src and the Mechanotransduction 

The involvement of cytoplasmic Src in the cell response to mechanical stimulation has been well characterized, especially in its crucial role of triggering the tyrosine phosphorylation cascade thought to be pivotal for mechanosensing [73]. Indeed, extracellular matrix proteins interaction with integrins induces their activation and the assembly of the focal adhesion complex proteins. This process, known as cell mechanotransduction, identifies involved proteins as mechanosensors, able to perceive and transduce mechanical stimuli into biochemical signals. Following integrin activation, the membrane-bound Src is responsible of an increase in focal adhesion kinase (FAK) and paxillin tyrosine phosphorylation, described as a first response to several mechanical stimuli, to such an extent that Src and FAK inhibitors are able to block the response to mechanical stimulation as the cyclic stretch [73]. 

In the context of mechanobiology, the Hippo pathway has been described to be relevant in regulating tissue growth and organ size [74,75]. The main function of the Hippo pathway is to inhibit Yes-associated proiein (YAP) and Tafazzin (TAZ) transcription co-activators, thereby regulating cell proliferation, apoptosis, and stemness in response to extracellular and intracellular signals, among which cell-cell contact, cell polarity, mechanical cues, ligands of G-protein coupled receptors and cellular energy status [75]. When YAP and TAZ are slightly phosphorylated they are more concentrated in the nucleus, thus leading to cell proliferation, wound healing or tissue regeneration [76]. Contrariwise, high levels of phosphorylation lead to cell quiescence [77]. It is also known that mechanical signals and phosphorylation can modulate YAP1 functions [78]. This may be related to Src-mediated phosphorylation of YAP1 in Tyr357 [79]. As a transcriptional factor, YAP1 is very important and two types of pathway are involved in its regulation: the “canonical” way (through the negative LATS1/2 regulation) and, as recently discovered, the SFK dependent way [80]. 

Ege and colleagues described for the first time the dominance of YAP1 nuclear export as the key point regulating its subcellular localization. Although serine phosphorylation is the first trigger required for YAP1 nuclear export, the inhibition of SFK activity by dasatinib in cancer related fibroblasts (CAFs) reduces the YAP1 nuclear localization leading to a higher citoplasmic content resembling normal fibroblasts. Indeed, CAF treatment with Src-family kinase inhibitors, such as dasatinib, affects the subcellular distribution of YAP1 by increasing the dissociation rate of YAP1 from chromatin thus inducing YAP1 export from nucleus. Among Src-mediated control of YAP1, its phosphorylation in Y357 functions as an independent mechanism for YAP1 activity regulation. Y357 phosphorylation seems to be not involved in controlling YAP1 subcellular localization, but in reducing its transcriptional competence. The evidence that YAP1 transcriptional activity is altered even when nuclear export is blocked suggests that this crucial phosphorylation may occur in the nucleus and that depends on nuclear Src activity [79]. 

Given the crucial roles of Src in the bone cells [20,23] and the great relevance of mechanical loads in the bone homeostasis [81], it is worth to mention the nuclear Src functions in osteoblast cells in response to mechanical stimulation. Indeed, external mechanical loads as the interstitial fluid shear stress are sensed at the membrane by integrins that transmit the message through ERK, Src and RhoA to actin stress fibers in the cytoskeleton [82]. Osteocytes, the most abundant cells of the bone tissue, reside into the mineralized matrix and are capable of sensing mechanical cues applied to the bone, to which they react triggering mechanisms involved in controlling osteoblast and osteoclast activities [83]. In particular, osteocytes respond to mechanical loading inducing the formation of a Src/Pyk2/MBD2 complex that suppresses anabolic gene expression [84]. Once activated by oscillatory fluid shear stress, Pyk2 and Src translocate into the nucleus, where they associate with methyl-CpG-binding domain protein 2 (MBD2), a protein involved in DNA methylation. Therefore, the formation of a nuclear Pyk2/Src complex in osteocytes is related to altered transcription and epigenome regulation, leading to the suppression of anabolic gene expression, likely a mechanism to prevent an over-reaction to physical stimuli [84]. 

## 5. Prognostic Roles of Nuclear Src

Beside the aforementioned functions of nuclear Src, its subcellular localization in tumoral cells has been associated to patient survival, being a useful prognostic factor. 

In our recent work, we described Src nuclear compartmentalization as a good prognosis factor for osteosarcoma patients’ overall survival as assessed by tissue microarray analysis [46]. Indeed, a high nuclear Src accumulation is detected in normal osteoblasts as well as in low-aggressive osteosarcoma cell line SaOS2 cells, while its nuclear localization decreases in relationship to tumor aggressiveness, being very low in high metastatic 143B cells. The regulation of the Src nuclear content in these cells seems to be related to its myristoylation status, having myristoylated Src a prevalent cytoplasmic localization. Indeed, the low NMT expression observed in low aggressive osteosarcoma cells can be related to a reduced myristoylation of many proteins, other than Src. It is worth noting that high levels of NMT expression have been associated to more aggressive tumors and NMT inhibitors are suggested as potential chemotherapeutic agents [85,86].

In sight of this, further studies are needed to confirm the close relationship among Src nuclear localization, the NMT expression and the osteosarcoma aggressiveness.

These results suggest that immunohistochemical analysis of Src subcellular localization, together with its expression, can provide more accurate information in the assessment of osteosarcoma prognosis [46]. 

In support of the prognostic relevance of nuclear Src in human tumors, Campbell and coauthors demonstrated that phosphorylated Src in the nucleus is also associated with improved patient outcome in estrogen receptor-positive tamoxifen-treated breast cancer [87]. 

This evidence seems to suggest that Src nuclear localization is associated to lower aggressiveness in cancer. Interestingly, the aforementioned works (the only two cases in the literature providing the prognostic relevance for Src subcellular localization) refer to osteosarcoma and estrogen receptor-positive breast cancer, being the former a bone tumor and the latter a cancer with high tropism to bone as its primary site of metastases [88]. 

Therefore, taken together, these works suggest peculiar Src nuclear functions in “bone-related” tumors as a sort of “physiological” role that need further investigation.

## 6. Conclusions

The Src family of tyrosine kinases exerts a plethora of roles inside the cell, both at a physiological and at a pathological level. In this review, we summarized the new emerging roles for Src recently described to be located in the nuclear compartment and to interact with nuclear proteins. Noteworthily, although the aforementioned works described a nuclear localization for Src, most of them did not provide evidence about the mechanisms responsible of the shuttling into the nucleus. Staring from the paper by David-Pfeuty et al., we speculated in this review about the importance of myristoylation status as a crucial point involved in Src subcellular localization, emphasizing how myristoylated proteins are anchored to the membrane, while the nuclear content of Src is the fraction of low-myristoylated proteins.

In the nucleus of normal and cancer cells, Src is involved in several activities involving both its enzymatic activity as tyrosine kinase and its capability to interact with other protein thereby forming protein complexes. In particular, Src participates in the regulation of chromatin reorganization and transcriptional activity of transcription factors, in modulating nucleoskeleton shape in response to mechanical stimulation by interacting with nuclear lamins and emerin, and it is surely involved in the oncogenic transformation of tumoral cells, by repressing some oncosuppressors. It is worth noting that Src nuclear functions can vary greatly depending on the type of assessed normal and/or tumor cells and they are not solely related to increased cancer aggressiveness. Indeed, in osteosarcoma and in hormone-positive breast cancer the Src nuclear compartmentalization is associated with improved patients’ overall survival. This evidence suggests a sort of physiological relevance for Src nuclear localization, confirmed by the high Src nuclear content observed in normal osteoblasts [46].

In summary, beside the well-known pivotal roles of Src and the other members of the family exerted in the cell cytoplasmic compartment, also its more recently recognized nuclear subcellular localization worth to be considered especially in the context of pathological conditions.

## Figures and Tables

**Table 1:** Subcellular distribution of Src-family kinases (SFKs) other than Src. CM: cell membrane; C: cytoplasm; N: nucleus.

SFKs	Subcellular Localization			References
	CM	C	N	
Yes	X	X		Dubois et al. [29]
Fyn	X	X	X	Saito et al. [30] - Matsushima et al. [31]
Fgr	X	X		Dwyer et al. [32]
Lck	X			Stephen et al. [33]
Hck	X	X		Poh et al. [34]
Blk	X			Petersen et al. [35]
Srm		X		Serfas and Tyner [36]
Brk	X	X	X	Derry et al. [37]
Lyn	X	X	X	Yoshida et al. [38]
Frk (Rak)		X	X	Ogunbolude et al. [39] - Kim et al. [40]

## Abbreviations

Blk	B Lymphoid tyrosine Kinase
Brk	BReast tumor Kinase
CAFs	Cancer Related Fibroblasts
Csk	C-terminal Src Kinase
ERα	Estrogen Receptor alpha
FAK	Focal Adhesion Kinase
Fgr	Gardner-Rasheed Feline Sarcoma Viral Oncogene Homolog
Frk	Fyn Related Src family tyrosine Kinase
Hck	Hematopoietic Cell Kinase
Lck	Lymphocyte-specific protein tyrosine kinase
Lyn	v-yes-1 Yamaguchi sarcoma viral related oncogene homolog
NADPH	Nicotinamide Adenine Dinucleotide PHosphate
NLS	Nuclear Localization Signal/Sequence
NMTs	N-myristoyltransferase
PRG	Progesterone
PTP	Protein Tyrosine Phosphatase
RTKs	Receptor Tyrosine Kinases
ROS	Reactive Oxygen Species
SAPK	Stress-Activated Protein Kinase
SFKs	Src Family Kinases
Shc	Src Homology 2 domain Containing
SHP2	Src Homology 2 containing protein tyrosine Phosphatase 2
Srm	Src-related kinase lacking C-terminal regulatory tyrosine and N-terminal myristylation sites
TAZ	Tafazzin
YAP	Yes-Associated Protein
Yes	Yamaguchi sarcoma viral oncogene homolog
